# Unite to predict

**DOI:** 10.7554/eLife.66223

**Published:** 2021-02-12

**Authors:** Meeraj Kothari, Daniel W Belsky

**Affiliations:** 1 Butler Columbia Aging Center, Columbia University Mailman School of Public HealthNew YorkUnited States; 2 Department of Epidemiology, Columbia University Mailman School of Public HealthNew YorkUnited States

**Keywords:** biological aging, telomere, epigenetics, metabolomics, transcriptomics, proteomics, Human

## Abstract

Integrating the analysis of molecular data from different sources may improve our understanding of the effects of biological aging.

**Related research article** Jansen R, Han LK, Verhoeven JE, Aberg KA, van den Oord EC, Milaneschi Y, Penninx BW. 2021. An integrative study of five biological clocks in somatic and mental health. *eLife* **10**:e59479. doi: 10.7554/eLife.59479

The molecular features of the human body change as we age, increasing the risk for disease, disability and mortality (Campisi et al., 2019). Omics technologies allow scientists to observe such changes – in, for example, levels of DNA methylation, gene and protein expression, or metabolites circulating in the blood – in large human populations. The resulting datasets are being mined to uncover the changes that occur as people age.

Two striking findings from these data-mining efforts have been: i) that reliable differences between older and younger humans exist at most molecular levels, (e.g. Bell et al., 2019; Johnson et al., 2020; Peters et al., 2015; Srivastava, 2019); and ii) that machine learning techniques can use these molecular datasets to construct algorithms, often referred to as ‘clocks’, that predict the chronological age of the individual from whom the sample was taken (Horvath, 2013; Lehallier et al., 2019; Robinson et al., 2020; Zhavoronkov and Mamoshina, 2019).

It has been proposed that errors in the predictions made by these clocks (that is, when the predicted age differs from the actual age) reflect the 'biological age' of the person. Biological aging is the gradual and progressive decline in system integrity that occurs with advancing age. More advanced biological ages correspond to increased physical deterioration leading to increased risk of age-related disability and mortality. An outstanding question is whether the prediction errors of different clocks reflect a common set of correlated biological processes, or if distinct aspects of aging impact different molecular features independently. Previously, no study of aging has brought together data from more than a few different molecular levels of analysis in a single sample. Now, in eLife, Rick Jansen (VU University Medical Center, Amsterdam) and colleagues – Laura Han, Josine Verhoeven, Yuri Milaneschi and Brenda Penninx (all from Amsterdam), and Karolina Aberg and Edwin van de Oord (both from Virginia Commonwealth University) – report an analysis of clocks developed from five different molecular features that provides a provocative answer to this question (Jansen et al., 2021).

Jansen et al. used machine learning methods to develop molecular clock algorithms based on whole genome DNA methylation, transcriptomics, proteomics, metabolomics and telomere length. The data they used came from blood samples taken from a cohort of young and midlife Dutch adults. The different clocks could predict the chronological ages of the participants with high accuracy, but the errors in the predictions differed significantly from clock to clock. This echoes findings from earlier studies comparing blood-chemistry and genomic approaches to the measurement biological aging (Belsky et al., 2018; Li et al., 2020).

The researchers also found that combining the outputs of the five clocks produced a summary score that was somewhat more predictive of participants’ health than the individual clocks. However, effect sizes for the individual clocks were very small (most r < 0.1), with the exception of moderate correlations between the metabolomic clock and obesity and metabolic syndrome (r ~ 0.2), and the composite score effect sizes were only slightly stronger (most r ~ 0.1; r ~ 0.3 for obesity and metabolic syndrome). Nevertheless, this result suggests that multi-omics phenotyping of human samples may be able to measure biological aging more precisely than approaches that rely on data from a single molecular feature.

Together, these findings are consistent with the hypothesis that different molecular features may record distinct aspects of biological aging. However, the small effect sizes reported for associations of different molecular clocks with aging outcomes (like disease and mortality) raise an important question: are age-correlated biological features necessarily features of biological aging? Put another way, can we measure biological processes of aging simply by identifying biological measurements that differ between older and younger people?

Clocks developed by identifying differences between older and younger individuals through data mining tend to be quite good at predicting chronological age in new samples. However, what matters more for studies of aging is understanding whether differences in the clocks’ predicted ages for humans of the same chronological age are due to differences in the progressive decline of their bodies that causes aging-related disease, disability, and mortality.

Efforts to develop measure of biological aging through data mining face two main challenges. First, age differences between participants correspond to differences in survival, what is known as survivorship bias. Only those who do not experience accelerated biological aging make it to older chronological ages. So, for chronologically older people, the only data available is that of survivors. The differences between this older population of survivors and the more complete population represented by the younger participants in the sample may conceal signs of aging (Nelson et al., 2020). Second, differences in biological markers between older and younger people may be independent of aging. For example, in cross-sectional studies, age is perfectly correlated with year of birth, which can confound differences in exposure to pathogens, environmental toxins, or nutrition with signs of biological aging (Moffitt et al., 2017).

One newer method to develop biological aging indices that protects against survivorship bias is to focus analysis on differences in mortality risk (Levine et al., 2018; Lu et al., 2019). Another method that addresses both survivorship bias and cohort effects is to model aging from changes that occur within individuals across repeated measurements taken over a period of their lives (Belsky et al., 2020).

The next step for multi-omics analysis of aging is to integrate new methods to control for survivorship bias and exclude cohort effects from measurements. The results of Jansen et al. suggest that multi-omics data can improve how biological aging is measured, and raise the possibility that theoretical models which define aging as a set of correlated processes that drive health decline may need to be revised. Further studies with multi-level molecular-level data will help to clarify both the potential for improved measurements and the implications for models of aging.
